# Evaluating cytotoxic effects of recombinant fragaceatoxin C pore forming toxin against AML cell lines

**DOI:** 10.22038/IJBMS.2018.26600.6516

**Published:** 2018-09

**Authors:** Mahnaz Azadpour, Maedeh Karimian, Mohammad Hassan Kheirandish, Abolghasem Asadi-Saghandi, Mehdi Imani, Akram Astani, Hossein Zarei Jaliani

**Affiliations:** 1 Protein Engineering Laboratory, Department of Medical Genetics, School of Medicine, Shahid Sadoughi University of Medical Sciences, Yazd, Iran; 2 Department of Advanced Medical Sciences and Technologies, Faculty of Paramedicine, Shahid Sadoughi University of Medical Sciences, Yazd, Iran; 3 Department of Biochemistry, Faculty of Veterinary Medicine, Urmia University, Urmia, Iran; 4 Department of Microbiology, School of Medicine, Shahid Sadoughi University of Medical Sciences, Yazd, Iran

**Keywords:** Acute myeloid leukemia, Fragaceatoxin C, HL-60 cell, KG-1 cell, Pore-forming toxin, Recombinant expression

## Abstract

**Objective(s):**

Current therapeutic strategies for cancer are associated with side effects and lack of specificity in treatments. Biological therapies including monoclonal antibodies and immune effectors have been the subject of multiple research projects. Pore-forming proteins may become the other biological strategy to overcome the problems associated with current treatments. But detailed mechanisms of their action on target membranes remained to be elucidated. We aimed to study the cytotoxic effects of recombinant form of fragaceatoxin C on AML cell lines HL-60 and KG-1.

**Materials and Methods:**

We cloned the FraC gene in pET-28a (+) bacterial expression vector and the expressed recombinant FraC protein was purified by affinity chromatography. Then, cytotoxic effects of the recombinant protein were examined on two AML cell lines, HL-60 and KG-1. Effects of serum and calcium ion were explored by hemolysis assay in more details.

**Results:**

Our results showed that the recombinant C-terminal polyhistidine-tagged FraC protein has potent cytotoxic effects on both AML cell lines, with IC_50_=5.6, and 4.6 µg.ml-1 for HL-60 and KG-1 cells, respectively. Serum showed dose-dependent and also time-dependent inhibitory effects on the hemolytic and cytotoxic activities of the FraC protein. Pre-incubation of the toxin with different concentrations of calcium ion also inhibited hemolytic activity of FraC toxin.

**Conclusion:**

Results of the present study showed that FraC has potential anti-tumor effects. By detailed investigation of the inhibition mechanism of serum and calcium effects in the future, it can be possible to design target sites for clinical applications of the toxin.

## Introduction

Acute myeloid leukemia (AML) is the most common form of myelogenous leukemia in adults. AML can occur in all ages, but more than half of the patients are older than 60 years. Therapeutic strategies for the treatment of AML include chemotherapy, radiotherapy, bone marrow and stem cell transplantation, biological therapy and surgery. While radiotherapy and chemotherapy has been shown to be more useful, both are associated with adverse side effects and disease recurrence. The main factors limiting the effectiveness of treatment are drug resistance, non-specific targeting and non-specific toxicity, which are still responsible for relapsed and resistant forms of the AML and its treatment-related mortality (1, 2). Latest advancements in AML treatment strategies including their promises and shortcomings and also novel therapeutic chemical drugs have been reviewed elsewhere (3). The main impediment to achieve effective AML therapies seems to be the discovery of agents with novel mechanisms of action especially with more specific targeting of AML blasts (4). Latest biological approaches including monoclonal antibodies and engineered immune effectors have many advances in preclinical research and clinical trials (5). Also fusion proteins capable of specific targeting of cancer cells have been the subject of multiple research studies (6, 7).

Pore-forming proteins and peptides may become the other biological strategy to overcome the problems associated with current treatments. They exert their toxicity by devastating the selective permeability of the cell membrane and finally disrupting membrane integrity. They comprise a large superfamily of proteins in a variety of organisms such as bacteria, insects, reptiles and biting marine invertebrates. These proteins are secreted in media as water-soluble toxins, but as soon as they reached to their target membrane, they changed to a membrane inserted form (8). 

Pore-forming toxins (PFTs) can be classified structurally into two groups: α-PFTs and β-PFTs (9). Surface of the α-PFT group has a three-layer structure consist of about 10 α- helix, while β-PFTs toxins are rich in β-sheets. Alpha helix and beta sheets create the central parts of the pore (10-12). Plasma membrane integrity is essential for the proper function and stability of living cells. Many pathogenic microorganisms secrete pore-forming proteins to create pores in the membrane of target cells. This will lead to permeability of the target membrane to different molecules and ions depending on the pore size. The activity of these pore-forming molecules is to disrupt membrane integrity by oligomerization in the membranes leading to collapse of the selective permeability of the membrane and cell death (13).

One of the representative families of eukaryotic toxins belonging to the α-PFTs are actinoporins. They are produced by different species of sea anemones as a single chain polypeptide consists of about 175 amino acids. These toxins have basic isoelectric point and usually a small amount of cysteine residues (14). A member of this family that has been recently isolated from the sea anemone *Actinia fragacea* is fragaceatoxin C (FraC) with molecular weight of 19.7 kDa and the PI of 9.75. FraC pores consist of a funnel-shaped symmetric particle, containing eight identical protein chains with the external diameter of 11 nm and height of 7 nm (15).

SPore-forming peptides with potent antimicrobial properties could be potential anti-tumor agents due to their lytic capacity. They are attracting more attention for using as anti-tumors and anti-parasites. It has been demonstrated that these toxins have strong cytolytic activity against mouse fibroblast cell lines and multiple cancer cell lines (16, 17). 

n the present study, we tried to assess cytotoxic activity of FraC toxin on HL-60 and KG-1 cell lines in order to analyze the cytotoxic activity of this toxin in more detail. Our preliminary studies showed that fetal bovine serum (FBS), calcium and some other cations have inhibitory effects on hemolytic activity of FraC and other PFTs. To apply PFTs *in vivo*, protein engineering and solvent modification strategies should be taken into consideration. For this purpose, we have investigated the inhibitory effects of serum and calcium ion on the hemolytic and cytotoxic activities of FraC in more details in the present study. 

## Materials and Methods

The *Escherichia coli* BL21 (DE3) competent cells were purchased from New England Biolabs (NEB). Restriction enzymes XhoI and NcoI were from Thermo Scientific. The human cell lines HL-60 and KG-1 were purchased from Pasteur Institute of Iran (Iran-Tehran). Standard protein marker, isopropyl β-D-1-thiogalactopyranoside (IPTG), T4 DNA ligase, plasmid extraction and DNA gel recovery kits were from Sinaclon (Iran-Tehran). The pET-28a (+) vector was from Novagen. All other chemicals were from molecular biology grade suppliers (Merck and sigma). 

***Gene amplification***

FraC coding sequence was ordered to be chemically synthesized with flanking NcoI and XhoI restriction sites. Gene was delivered into pUC57 plasmid. Empty pET-28a vector and the pUC57 harboring FraC gene were double digested by the two restriction enzymes for 1 hr at 37 ^°^C. The resulting fragments of FraC were cut from the gel, purified and sub-cloned into digested pET-28a. Recombinant construct was named pET28a-FraC. *E. coli* DH5α and *E. coli* BL21 (DE3) competent cells were prepared by calcium chloride (CaCl₂) method (18) and were used for transformation of recombinant construct. The plasmid pET28a-FraC was transformed into competent *E. coli* DH5α as the primary host in order for amplification of recombinant plasmid and subsequently into *E. coli* BL21 (DE3) cells as the expression host for recombinant protein production using kanamycin resistance for selection (18). 

***FraC expression and purification***

Expression of recombinant FraC was carried out in *E. coli* BL21 (DE3) cells. Briefly, 1 ml of the overnight culture of recombinant *E. coli* BL21 (DE3) grown in Luria Bertani broth containing 0.1 mg.ml^-1^ kanamycin was transferred to 100 ml of the same medium. This culture was grown at 37 ^°^C until an A^600 nm^ reached 0.6 - 0.8. Protein expression was induced by adding 0.5 mM IPTG overnight, and cells were harvested by centrifugation at 6000 rpm for 10 min. The cell pellet was then resuspended in the minimum volume of lysis buffer (Tris 200 mM, pH 8.0, NaCl 300 mM, and Imidazole 5 mM) containing 4 mM dithiothreitol (DTT). Afterward, the resuspended cells were disrupted by sonication (70 kHz, on ice) and centrifuged at 12000 rpm, at 8°C for 20 min. The supernatant of the lysed bacteria was applied onto a Ni^2+^-NTA column. The column was pre-equilibrated and after applying the supernatant was washed with the same buffer as lysis (except of the 10 mM imidazole). Fractions were eluted using elution buffer (the same as lysis except of the 300 mM imidazole). Aliquots of the fractions were analyzed with sodium dodecyl sulfate-polyacrylamide gel electrophoresis (SDS-PAGE) and the fractions containing the protein with desired molecular weight were dialyzed against storage buffer (Tris 100 mM, pH 8.0, NaCl 50 mM, and glycerol 10%).

SDS-PAGE was carried out as described by Laemmli (19). Whole cell lysates were collected before and after induction and were examined by SDS-PAGE 17.5%. Protein staining was carried out with Coomassie Blue dye. Low-range molecular weight markers were used as reference.

***Hemolytic activity***

Human fresh red blood cells (RBC) were washed with 10 mM PBS buffer (137 mM NaCl, 2.7 mM KCl, 10 mM Na_2_HPO_4_, 2 mM KH_2_PO_4_, pH 7.4) until the supernatant was clear. The erythrocytes were resuspended in the same buffer and the A^600 nm^ was adjusted to 1.50. To determine hemolytic activity of FraC toxin, we carried out 2-fold serial dilutions of purified recombinant FraC in PBS buffer. Afterwards, 20 µl of each dilution of the FraC was added to a total 500 µl of a suspension of human erythrocytes. After an incubation period of 10 min at 37 ^°^C, A^600 nm^ was measured by spectrophotometer.

To assess the effect of serum on hemolytic activity of the purified FraC toxin, aliquots of the purified FraC protein was subjected to dilute in FBS (from Gibco^®^). Incubation of the toxin and FBS was performed at different times before measuring the hemolysis activity of the toxin. In an independent experiment to determine the effect of heating on the serum effect, ½ dilutions of two fractions of the FBS (intact and heated in 95 ^°^C for 5 min) was incubated for 5 min with aliquots of toxin, and the hemolytic activity of the toxin was analyzed as mentioned above.

Similarly, the effects of CaCl_2_ were assessed by pre-incubating a constant concentration of FraC toxin (12.5 µg.ml^-1^) with increasing concentrations of CaCl_2_. After 5 min of incubation, the hemolytic activity of the toxin was analyzed as mentioned above. For the study of the effects of CaCl2 and FBS on the hemolytic activity of the toxin, a dilution of the toxin with 50-100 % hemolytic activity was used. So both the resultant increase and decrease of the toxin hemolytic activity could be followed on the graphs.

**Figure 1:**
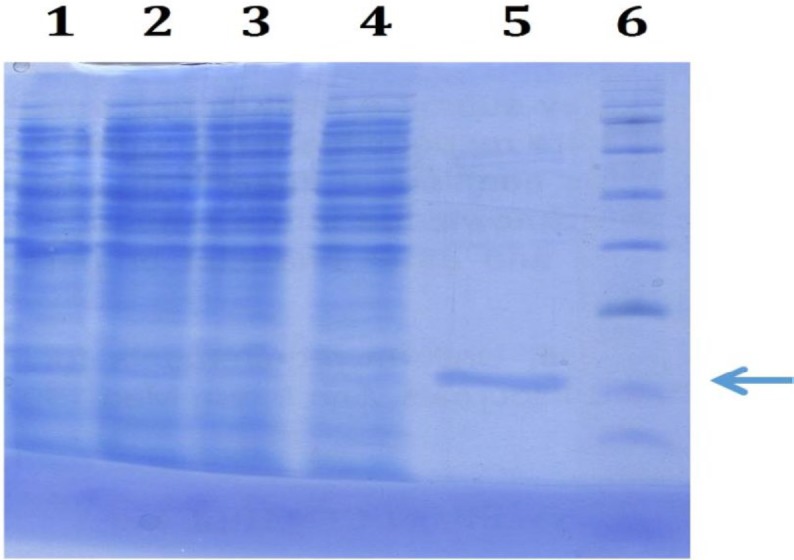
Purification of the fragaceatoxin C toxin using 17.5% SDS-PAGE. Lanes 1-4 are soup of the sonicated bacteria, pellet, flowthrough, and wash of the column. Lane 5 is the first eluted fraction and lane 6 is the protein size marker. Purified fragaceatoxin C protein band was demonstrated by arrow

**Figure 2:**
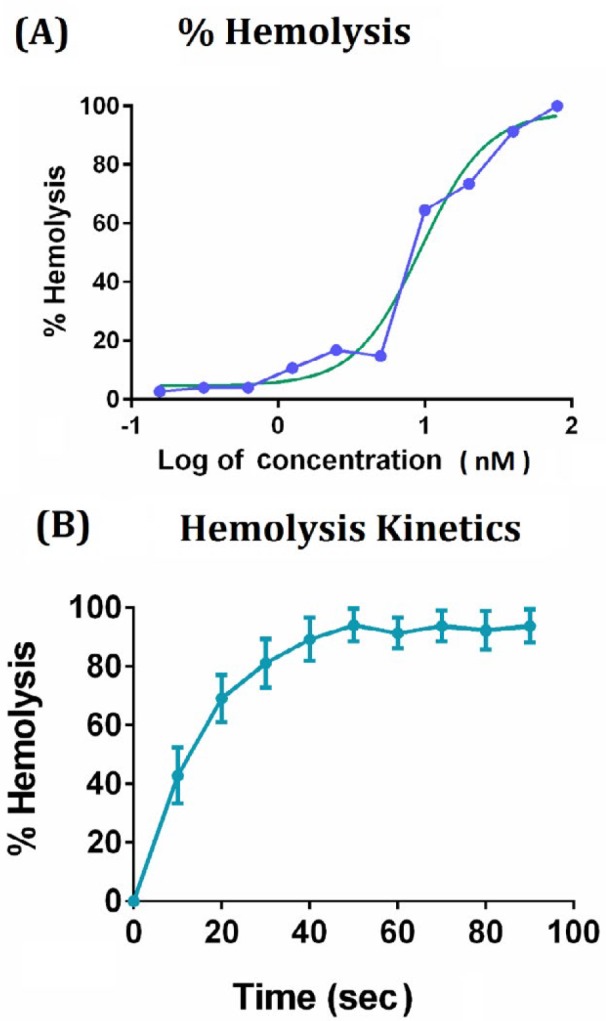
Hemolysis activity of purified fragaceatoxin C toxin. Hemolytic activity of different concentrations of the fragaceatoxin C toxin was assayed (A). Hemolysis of the human RBC was kinetically monitored after addition of the ¼ dilution of the fragaceatoxin C toxin (B)

**Figure 3:**
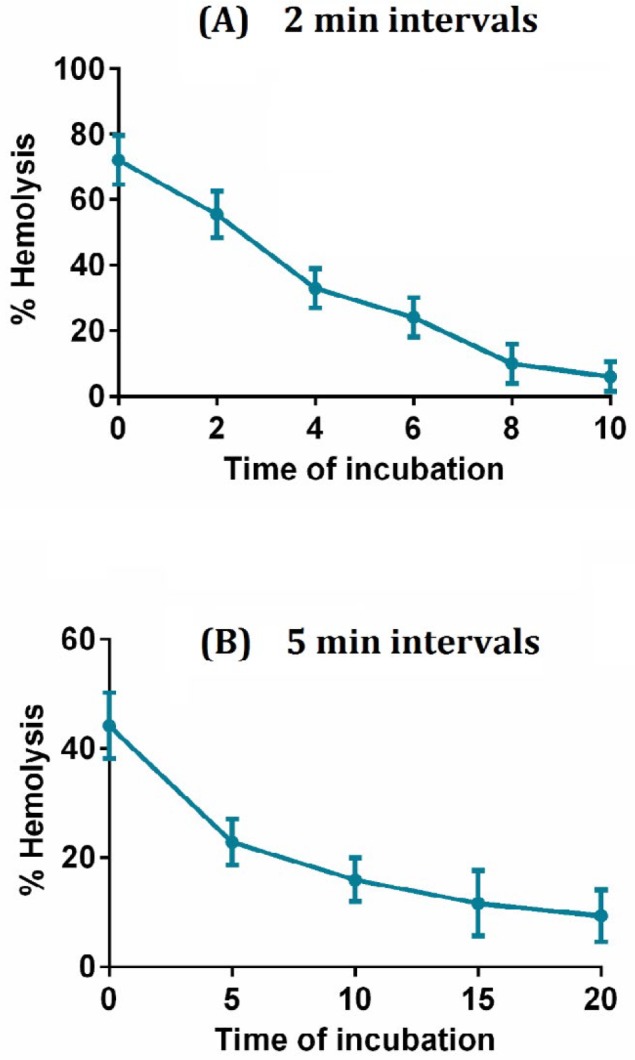
Effect of serum incubation on fragaceatoxin C hemolytic activity. Fixed dilutions of the fragaceatoxin C toxin was incubated with FBS at different time periods, 2 min intervals (A) and 5 min intervals (B)

**Figure 4:**
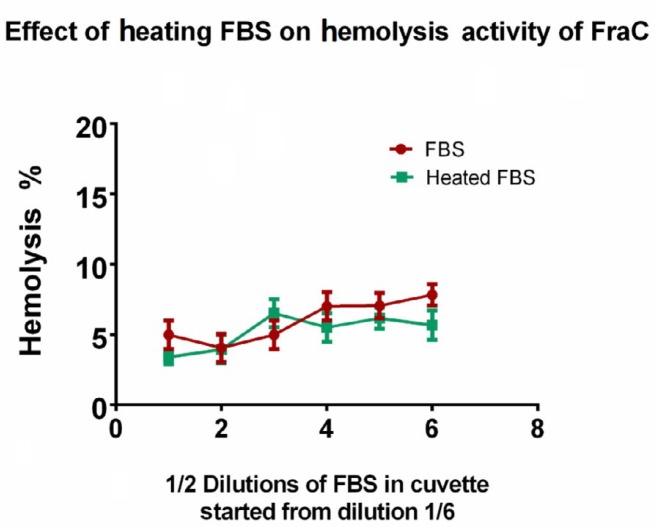
Effect of heating on inhibitory effect of serum on fragaceatoxin C hemolytic activity. Serial dilutions of the two fractions of FBS (intact FBS and heated at 95 °C for 5 min) were treated with a fixed concentration of fragaceatoxin C toxin and its hemolytic activity was measured after 10 min of incubation with FBS dilutions

**Figure 5:**
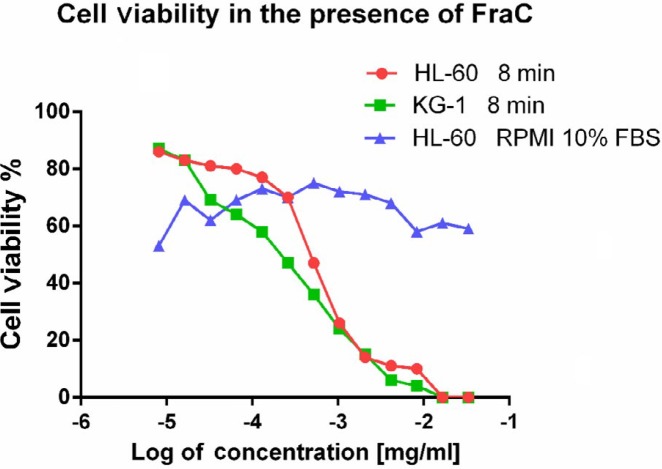
Cell viability of HL-60 and KG-1 cell lines in the presence of fragaceatoxin C toxin. Serial dilutions of the purified fragaceatoxin C toxin was treated with HL-60 and KG-1 cells in RPMI-1640 media supplemented or not with 10% FBS

**Figure 6:**
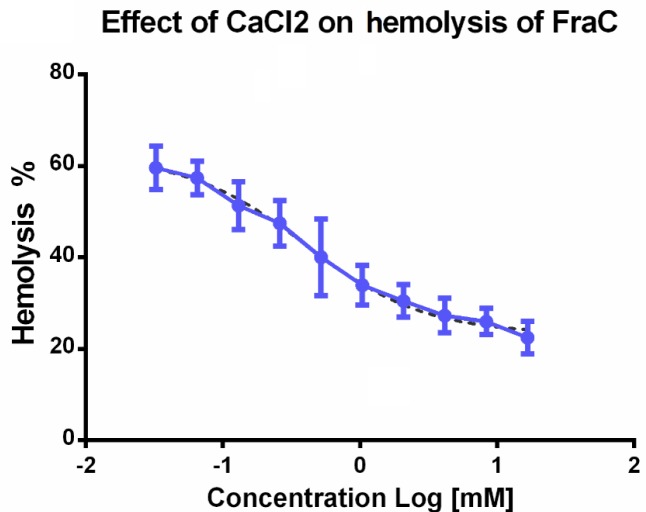
Effect of CaCl2 on hemolysis activity of fragaceatoxin C toxin. fragaceatoxin C toxin was incubated with different concentrations of CaCl_2_ and the hemolysis activity of the incubated toxin was determined after 10 min

***Cell culture and viability assays***

Two human cell lines, HL-60 (Human promyelocytic leukemia cell) and KG-1 (Human myeloid cell line) were cultured in RPMI 1640 medium, supplemented with 10% FBS at 37ºC in humidified 5% CO_2_.

Cytotoxic effects of recombinant FraC protein were determined using trypan blue assay. HL-60 and KG-1 cell-lines were harvested by centrifugation at 2000 rpm for 5 min. Afterward, the supernatant was discarded, and the cells were washed with PBS and centrifuged three times at 2000 rpm for 5 min. Pellets were resuspended in 700 µl PBS. 10 µl of 2-fold serial dilutions of recombinant FraC in PBS buffer were prepared, and then 50 µl of the resuspended cells (1-2×10^6^ cell.ml^-1^) were added to each tube and after 5 min of incubation at 37 ºC, cell viability was determined by adding 50 µl trypan blue (%0.4 w/v) to each tube using equation below. All experiments were repeated three times. *P* values were used to determine statistical significance. 

(%) Cell viability =×100

## Results

***Protein expression and purification***

After sonication of the harvested induced bacteria and passing through Ni-NTA column, FraC protein was purified at the yield of 4.5 mg.l^-1^ with the purity of about 80% (as determined by ImageJ software) as demonstrated in Figure 1, as a single band on SDS-PAGE. 

***Serum inhibitory effect on hemolytic activity of FraC***

Serial dilutions of the purified FraC toxin were prepared and the hemolytic activity of the fractions was monitored after 10 min of incubation with 5% human RBC suspension. As illustrated in Figure 2A, hemolysis activity of the toxin was dose-dependent and its IC_50_ was measured to be 8.9 nM (as calcuated by Graphpad Prism ver. 11). Activity of the FraC toxin was very rapid since the ¼ dilution of the purified toxin completely lysed the RBC solution in 1 min (Figure 2B). 

As shown in Figure 3, FraC toxin loses its hemolytic activity after pre-incubation with FBS. By increasing the time of FraC incubation with serum, the all activity has been lost. This result shows that there might not be a competing agent in the serum, which inhibits the activity of FraC toxin.

As shown in Figure 4, there is no statistically significant difference between intact FBS and heated FBS. The rational for this assay was that if there were any proteinaceous fractions in FBS that inhibits hemolytic activity of the FraC by such an enzymatic reaction (e. g. any kind of proteases), so the heating could reverse the inhibitory effect of the serum. As the results show, it seems that there is not any enzymatic activity responsible for the inhibitory effect of the serum. 

***Serum inhibitory effect on cytotoxic activity of FraC***

Several cell viability experiments in our laboratory have shown that FraC toxin has not activity against cell membranes in the presence of FBS. As shown in Figure 5, HL-60 and KG-1 cells were completely lysed (permeabilized to trypan blue) in the presence of higher concentrations of FraC toxin only if they had been resuspended in serum-free RPMI media or PBS buffer. In the presence of serum, not only the activity of the FraC toxin was orders of magnitude lower, but it was not dose-dependent too. The IC_50_ of FraC for HL-60 and KG-1 cell lines were 5.6, and 4.6 µg.ml^-1^, respectively. 

***Effect of calcium on hemolysis activity of FraC***

As shown in Figure 6, calcium chloride could apparently inhibit the hemolytic activity of the FraC toxin. IC_50_ of the inhibitory effect of calcium ion was determined to be 0.2 mM, which is in the range of sub-physiological concentrations of calcium ion. 

## Discussion

Pore-forming proteins are found in many pathogenic bacteria and in some vertebrates. They have similar structures and also share similar mechanisms in permeabilizing cell membranes to water molecules and solutes. Bacteria use these proteins as virulence factors and vertebrates may use pore-forming proteins as part of their immune systems (20). Pore-forming proteins insert into cell membranes and oligomerize into pore structures ranging in size from 1-2 nanometers in anemone cytolysins (21) to over than 30 nanometers in cholesterol-dependent cytolysins (CDCs) (22). 

Although it has been shown that these proteins have potential to kill tumor cells, many attempts in this area has been limited due to the lack of specificity of the toxin molecules for the cancer cells or the lack of detailed information about the killing mechanisms (23-26). 

In the present study, we have expressed the recombinant form of FraC from strawberry anemone (*A. fragacea*) in *E. coli*. After purification of the toxin with Ni-NTA affinity chromatography, hemolytic and cytotoxic activities of the toxin have been explored in more details. 

Hemolytic activity of the FraC had been previously explored using the native protein extracted from the anemone itself and also in the form of a recombinant histidine-tagged protein. In the present study, IC_50_ of the recombinant polyhistidine-tagged FraC in human RBC hemolysis assay was shown to be 8.9 nM comparable to the native protein extracted from *A. fragacea *(27, 28) and the other previously studied recombinant form (29). 

The importance of N-terminal in the actinoporin family of PFTs has been the subject of previous studies. It has been shown that the N-terminal amino acids of equinatoxin II are very important in toxin oligomerization and consequently pore formation in target cell membrane (30, 31). In this study, NcoI restriction site of the pET-28a (+) expression system was used to have minimal modification of the N-terminal of the recombinant protein (only one Met was added to N-terminal of FraC protein). Also, the XhoI restriction site has been used to have minimal number of amino acids in the affinity tag on the C-terminal of the protein. Residues added to the C-terminal were as follow; [Leu-Glu-(His)6]. Of course it will be more interesting if the FraC toxin could be cloned without any additional amino acids other than affinity tag like streptolysin O in a recent study (32) to explore if there is any difference in IC_50_ of the recombinant proteins. But, the data of the present study shows that any impact due to the additional amino acids in the C-terminal on hemolytic activity of FraC protein is unlikely. 

Cytotoxicity results of the present study using trypan blue assay show that recombinant FraC has potent anti-tumor activity against both HL-60 and KG-1 cell lines. These results were comparable to cytotoxic activity of other actinoporins on leukemic cells and RBCs (16, 33).

Our results showed that serum (FBS) could effectively inhibit the cytotoxic activity of FraC on both cell lines. We have explored this effect in more details using the hemolytic activity of the toxin in the presence of FBS. It was shown that incubation of the FraC with FBS inhibited the hemolytic activity of the toxin and this effect was dependent on the incubation period. One possibility of the time-dependency of the serum effect was that there might be an enzymatic activity (e. g. protease activity) in serum, which affects the FraC molecules so that they cannot oligomerize or insert into the cell membrane anymore. Heating the FBS could not reverse the inhibitory effect of serum and this result showed that there is not enzymatic activity responsible for the inhibitory effect of FBS. Alternative possibility is that the high concentration of proteins in serum can aggregate FraC monomers or denature them so that they cannot insert into cell membrane anymore. If a specific site of interaction of any part of serum with FraC molecule is recognized and discovered, it is possible to engineer or remove it to produce toxins with the ability to lyse target cells in the serum environment. 

IC_50_ of the inhibitory effect of calcium ion was determined to be 0.2 mM, which is in the range of sub-physiological concentrations of calcium ion. Because the inhibitory effect of calcium on hemolysis activity of FraC was occurred at pre-incubating the toxin before adding the combination on RBCs, there might be a structural alteration in the FraC itself, which can be responsible for the effect of calcium. There is no similar report on any PFTs, and the effect of a range of ions on the structure and function of the FraC will be necessary at the future to elucidate the detailed profile of its structure-function relationship.

## Conclusion

PFTs could have various applications in research and therapeutics. Detailed knowledge on their mechanism of action and structure-function relationship is necessary to implement this exciting class of protein toxins in numerous applications. If inhibitory effect of serum studied here will be illustrated in more details in the future, and especially with respect to associated target sites on the FraC structure, it will be possible to modify the inhibition. The present findings might have important implications for *in vivo* use of FraC and also other PFTs. Future studies on the inhibitory effects of serum and physiologically important ions are therefore required in order to establish therapeutic approaches using PFTs. 
